# Animal-Assisted Activities for Patients with Central Nervous System Disease in Acute Rehabilitation Setting

**DOI:** 10.3390/brainsci15101029

**Published:** 2025-09-23

**Authors:** Vittorio Casati, Valentina Re, Paola Bardasi, Andrea Contini, Pilade Cortellazzi, Angelica Gallarati, Emilia Bozzini, Valentina Castignoli, Gianfranco Lamberti, Fabio Razza, Simona Galante, Chiara Frati, Francesca Ronchetti, Monica Morelli, Emanuela Ricci, Gianluca Ciardi

**Affiliations:** 1 Azienda Usl of Piacenza, 29121 Piacenza, Italy; v.casati@ausl.pc.it (V.C.); valentina.re@unipr.it (V.R.); p.bardasi@ausl.pc.it (P.B.); a.contini@ausl.pc.it (A.C.); p.cortellazzi@ausl.pc.it (P.C.); a.gallarati@ausl.pc.it (A.G.); e.bozzini@ausl.pc.it (E.B.); v.castignoli@ausl.pc.it (V.C.); g.lamberti2@ausl.pc.it (G.L.); f.razza@ausl.pc.it (F.R.); s.galante@ausl.pc.it (S.G.); c.frati@ausl.pc.it (C.F.); emanuela.ricci@unipr.it (E.R.); 2 Degree Course in Physiotherapy, Piacenza Training Centre, Department of Medicine and Surgery, University of Parma, 43125 Parma, Italy; 3 La Collina Dei Ciuchini-Vernasca (Piacenza), 29010 Vernasca, Italy; francesca.ronchetti@gmail.com (F.R.); monicamorelliistruttore@gmail.com (M.M.)

**Keywords:** animal assisted activities, animal assisted intervention, neuro-behavioral disorders, spinal cord injury, stroke, traumatic brain injury

## Abstract

**Background:** Animal-assisted activities (AAA) are participative interventions, designed to lower hospitalization-related stress and anxiety, enhance communicative readiness, improve quality of life and encourage human–animal interaction. The aim of the present study was to evaluate AAA effects in the context of intensive rehabilitation of patients with spinal cord injury (SCI), traumatic brain injury (TBI), stroke. **Methods:** AAA in this study were structured by a local specialized association, for small groups of patients (5/7 a time), biweekly; each session lasted 60 min. Each patient participated in 10 sessions of AAA. Evaluation rating scales were administered at T0 (before the first session) and T1 (after the last session, five weeks later) as follows: Neurobehavioral Rating Scale (NRS) in case of patient with stroke/TBI without disorder of consciousness; Hospital Anxiety and Depression Scale (HADS) for SCI patients. **Results:** A total of 50 patients concluded the study. NRS score for joined TBI and stroke populations varied from a T0 mean value of 32.34 [C.I. 26.83–37.35] to 17.21 [C.I. 12.66–21.76] (46.7%); this difference proved to be statistically significant (*p* = 0.000). Stroke patients had a 57.6% NRS lowering by mean 28.10 [C.I. 20.55–35.65] points to 12 [C.I. 6.6–17.36], which was significant (*p* = 0.000); similarly, TBI patients showed a mean decrease of 35.8% points from the initial 41.6 points [C.I. 37.29–45.93] to 26.76 [C.I. 21.94–31.59] (*p* = 0.002). As for HADS scores a smaller improvement was found in the cohort of SCI patients: anxiety registered a 1 mean point decrease at T1 (21.5%), from the initial 6.5 points [C.I. 3.80–9.34] to 5.1 ones [C.I. 3.17–7.11]. This variation was near the threshold of significance (*p* = 0.05). Depression domain, instead, improved by 2.35 mean points (37%), from the 6.35 initial points [C.I. 3.45–9.26] to the final 4 [C.I. 2.15–5.98] with reaching of a significant *p* value (*p* = 0.03). ANCOVA did not confirm this last value and showed no influence of age and gender on outcome variations. **Discussion:** AAA showed preliminary evidence to decrease neurobehavioral disorders in patients with high-complexity neurological diseases, particularly stroke and TBI. The role of AAA in SCI patients remains unclear. Future studies should address confounders’ role for these populations, particularly severity of disease. Furthermore, AAA interventions will have to be studied on larger samples, deepening the exact phase to introduce AAA for neurological patients. Lastly, qualitative studies are needed to explore patients’ lived experiences.

## 1. Introduction

Accounts of animal-based interventions for therapeutic purposes are recurrent throughout the course of human history; formal research into the use of animals for therapeutic purposes started in the 1960, when Boris Levinson, a child neuropsychiatrist, developed the theory of “pet-oriented child psychotherapy,” which is based on elements of child psychology and child–animal relationships [1]. Levinson investigated the use of pet psychotherapy for treatment of behavior disorders in children and, in 1961, published the results in his book “The Dog as Co-Therapist” [2]; the concept of “pet therapy” appears to have been first enunciated here.

More recently, different species of animals have been involved in a variety of animal-assisted intervention (AAI), such as dogs, cats, donkeys, horses and rabbits. To date, AAI consists of a wide range of activities aimed at improving health and well-being in people with the help of pets [3]; they are widely recognized in the literature as therapeutic, rehabilitative, educational and recreational tools. In Italy, the Ministry of Health, in order to promote research and standardize operational protocols, published the “National Guidelines for Animal-Assisted Interventions (AAI)” in 2015. These guidelines regulate how assisted interventions are delivered and classify them into three different areas, respectively, with therapeutic (animal-assisted therapy, AAT), educational (animal-assisted education, AAE) and playful–recreational and socialization values (animal-assisted activity, AAA) declining in practice differently one from each other and with distinct and only partly overlapping aims [3]. AAI are applied in a wide group of conditions, ranging from dementia [4], mental disorder [5,6], ICU settings [7], cancer [8], frailty [9] and heart disease [3,10]; application of AAI in pediatric populations is also highly represented in the literature [11,12,13].

AAA consist of a series of activities involving less structured human–animal interaction than AAT. They are frequently offered in small groups in order to reduce hospitalization-related stress and anxiety, enhance relational and communicative attitude, improve quality of life and encourage human–animal interaction [7,14,15]. An AAA-based program usually involves multidisciplinary teamwork, aiming for the following: defining activities according to the context and patients; conducting the program; reporting program effects [3].

The scientific literature highlights the role of AAA in improving emotional support and well-being of patients/families/health care staff in intensive care settings, while in post-acute wards the relationship with a dog can reduce pain, fatigue, stress and anxiety [4,15,16]. Despite the lack of knowledge about the physiologic mechanism induced by AAA, the literature argues that constant interaction with an animal increases social and mental well-being, decreasing the level of stress and depression in the patient [14,15]. In pediatric hospital wards, AAA increase children’s level of pleasure and ability to participate [15]. In patients with dementia AAA showed significant effects on quality of life and depression [4,17]. AAA have shown a strong positive effect on social behaviors, physical activity and food intake in dementia patients and a positive effect on reducing agitation/aggressiveness [4]. Although evidence exists to support the benefits of AAA in care settings, on both adult and pediatric populations, evidence is lacking regarding AAA effects in intensive neurorehabilitation inpatient setting. So, the aim of the present study was deepened to the following research question (RQ):

- RQ1: What is the effect of AAA protocol in patients with central nervous system disease with high rehabilitation needs?

From this perspective, the primary study end point regarded variations in Neurobehavioral Rating Scale (NRS) and Hospital Depression and Anxiety scale (HADS).

## 2. Materials and Methods

A prospective, single-cohort interventional study was conducted at the U.O. “Spinal Unit and Neurorehabilitation” of Fiorenzuola d’Arda Hospital, over a 1-year recruitment span. The study was approved by AVEN ethics committee (protocol num. 2023/0115453 dated 15 November 2023) and by Piacenza AUSL corporate management (by resolution 2023/0000536 dated 23 November 2023). The present study was conducted in conformity with the Strobe statement.

A multidisciplinary research team for this investigation was established, involving Physical Medicine and Rehabilitation specialists, Physiotherapists, Psychologists, Animals’ certified coadjutors of a local association (La Collina dei Ciuchini).

A convenience sampling strategy was conducted among patients admitted at Fiorenzuola d’ Arda Hospital from November 2023 to November 2024.

Sample size was calculated a priori using the G*Power software (version 3.1.9.7) for a paired sample *t*-test (two-tailed), with a significance level α = 0.05, a statistical power of 80% (1 − β = 0.80) and a mean expected effect (dz = 0.5). The calculation indicated the need to recruit at least 45 participants.

After defining inclusion/exclusion criteria, operators in the operative unit involved in the study (different by research team members) were asked to indicate potential eligible patients; after this first screening, a research team member proposed participation in the study. If the patient agreed, a short meeting was scheduled, during which the patient could sign informed consent; finally, two research team members (psychologist/Neuropsychologist and Physical Medicine and Rehabilitation specialist, not involved in conducting the AAA) performed evaluations. Inclusion criteria for participation to the study were as follows:

- Major age/minor age with consent of parent or legal guardian.
- Positive history of brain injury/stroke/spinal cord injury.
- Clinical stability expressed by the ward physician.
- Consent to adhere to the proposed activity.

Exclusion criteria:

- Allergy to cat hair.
- A-responsive waking state.
- Clinical instability.
- Bed resting with exclusion of load on the spine.
- History of phobia to dog or cat.
- Contact infections with indication for room isolation.
- Refusal to participate in the study.
- Severe neurological deficit of higher cognitive functions (neglect, aphasia, apraxia, dementia) due to impossibility to answer staircase questions.

All included patients underwent the following assessment:

1. General demographic data (sex, age, diagnosis, department of provenience, occupation); moreover, all patients underwent functional status assessment using the Modified Barthel Index, and the Shah et al. [18] classification was applied to identify the patients’ functional levels.
2. Neurobehavioral Rating Scale (NRS) for assessment of neuro-cognitive deficit in stroke/severe brain injury patient without disorder of consciousness. NRS is a clinician-rated questionnaire evaluating neurobehavioral disturbances such as agitation, emotional distress and cognitive deficits, with higher scores indicating worse neurobehavioral status [19].
3. Hospital Anxiety and Depression Scale (HADS), for the assessment of anxiety and depression, was administered to patients with spinal cord injury [20]. HADS is a self-administered 14-item questionnaire, developed to assess anxiety and depression in adults, particularly in hospital and community settings, with higher scores indicating mood compromise.

NRS and HADS were administered before participation to the study (T0) and at the term of tenth AAA session (T1), five weeks later.

AAA were delivered continuously for a year with a biweekly frequency; each session was conducted in a hospital meeting room and included 5–7 patients. Every session lasted 60 min and should focus on interaction with dog or cat (alternately); each patient participated in 10 sessions of AAA.

Based on patients’ level of interaction and propensity to interact, the animal coadjutor should propose the following activities to all patients during each session:

- Playful and interactive activities with structured material (balls, hoops, sensory mats).
- Walk with the dog.
- Activities involving purposeful physical contact with the animal (brushing, stroking).
- Petting activities with the cat lying on the patient’s legs in the wheelchair.
- Throwing the ball with request to return.

All patients who took part in the study continued daily standard rehabilitation treatment (1.5 h/day sessions with a physiotherapist, autonomous stretching or strengthening activity) activity alongside AAA sessions.

Statistical analysis: Data were organized into excel worksheets and then analyzed using SPSS 20.0 software. We identified changes in HADS and NRS scores as variables of interest, so confronting pre/post values (T0 vs. T1) of each patient (paired test) was performed to verify changes’ significance. Regarding NRS, we conducted the study on the whole stroke/TBI population, and then sub-analysis for single disease was performed; HADS was evaluated in SCI population according to its sub-sections (anxiety and depression).

Shapiro–Wilk and Kolmogorov–Smirnov tests were applied to assess normality of NRS and HADS distribution, then paired *t*-test was performed to assess difference significance for normally distributed data, and Wilcoxon paired test for not-normally distributed data. The level of significance was set for *p* < 0.05.

To further decrease the impact of baseline differences, a covariance analysis (ANCOVA) was performed on global scores of NRS and HADS, taking into account sex and age as possible confounders in results interpretation.

## 3. Results

**Figure 1:**
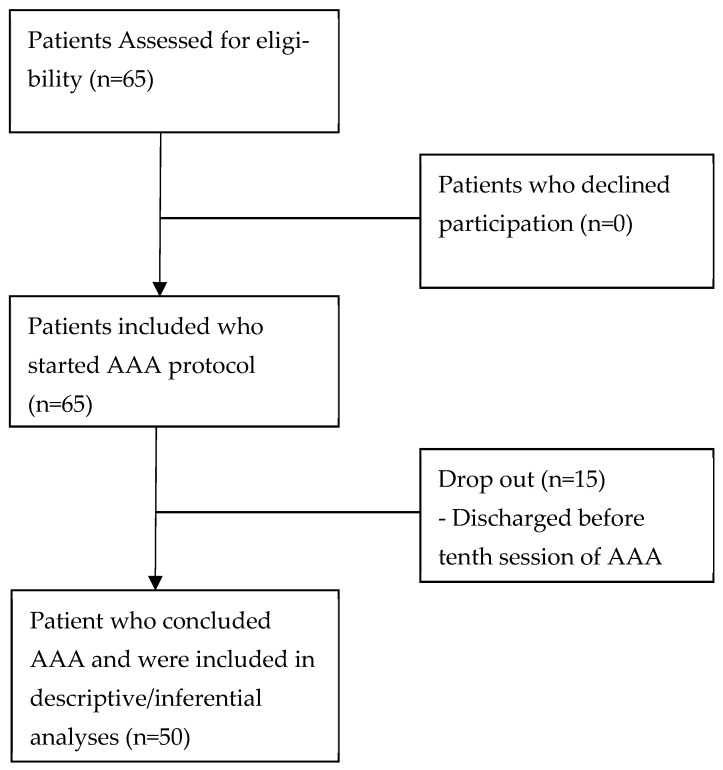
Flowchart of inclusion and participation to the study in accordance with Strobe guidelines.

The process of selection and inclusion of patients was described in Figure 1: the first study phase saw the screening of 65 potential eligible patients; of these, all presented inclusion criteria and agreed to participate. After the first assessment at T0 with evaluation scales (NRS or HADS), all patients started AAA sessions; during the study there were several dropouts (n = 15) due to discharge before the tenth AAA session (minimal number of sessions according to study protocol); so, finally, data of 50 patients were recorded at T1 (final evaluation) for descriptive and inferential analysis. No adverse events (such as bites, allergic reactions, or patient injuries) were reported during the AAA sessions.

In Table 1 a summary of principal socio-demographic data of our sample was reported. During the recruitment phase (that lasted 9 months) majority of included patients were males (40), while 25 were women. Regarding age, the mean overall value was 63.13 ± 16.706 years; as for subgroups a higher mean value was reported for TBI (mean 64.18 ± 16.70 years), while stroke (63.38 ± 17.85 years) and SCI patients (56.68 ± 17.58 years) were generally younger.

Regarding stroke patients, the main diagnosis was ischaemic stroke (25 cases); among TBI patients (15 cases), no one presented a disorder of consciousness (minimally responsive state, vegetative state, locked-in syndrome or coma). Regarding SCI patients (22 cases), there was a prevalence of not-traumatic forms (due to degenerative myelopathy, complication of lumbar stenosis, infective spinal disease), while 10 patients suffered a car/motor accident.

At the start of the study, most of the sample was admitted to Fiorenzuola Hospital for an intensive rehabilitation treatment from an emergency department (39 patients), while 11 arrived from the local trauma center; the minority of the sample was admitted by a further rehabilitation unit (7 patients) or by home (SCI patients who had indication of a brief intensive rehabilitation period). As for functional state, finally, all patients were evaluated through Modified Barthel Index (MBI) by a nurse at admission: accordingly to the Shah et al. classification [18] the majority of our sample (40 patients—62%) was composed by patients with a moderate functional compromission (BI 61–90), mainly in the area of locomotion/stairs climbing and autonomous upper limb use, while a small proportion had a severe compromission (BI 21–60, 20%). Lastly, 10 patients had a slight functional impairment (BI 91–99) and 2 were autonomous.

### Statistic Test

NRS and HADS variations were identified as variables of interest; particularly, we examined the global variation in NRS score for all patients, and within the subpopulation of stroke and TBI. Regarding HADS, the sub-study of anxiety and depression scores was conducted.

At Shapiro–Wilk and Kolmogorov–Smirnov tests, all T0–T1 scores were normally distributed, except NRS sub-analysis for stroke and TBI population. So, Wilcoxon paired test was performed for these last two comparisons, while paired *t*-test was used for the other ones. As shown in Table 2, all values showed a general trend of improvement following the treatment, both for general sample and for single populations. Particularly, NRS score for joined TBI and stroke population (range 0–91 points) varied from a T0 mean value of 32.34 [C.I. 26.83–37.35] point to 17.21 [C.I. 12.66–21.76], thus defining a consistent lowering of neurobehavioral disorders (46.7%); this difference proved to be statistically significant (*p* = 0.000). ANCOVA confirmed a statistically significant difference in pre–post NRS scores (F = 6.254, *p* = 0.018), controlling for age and gender. There was no significant interaction between time and gender (*p* = 0.580)/age (*p* = 0.97), suggesting that improvement following AAA was consistent across the study group.

Even regarding subpopulations an improvement was noted: stroke patients had the best mean NRS lowering by mean 28.10 [C.I. 20.55–35.65] points to 12 [C.I. 6.6–17.36], (16.1 points decrease, 57.6%), which was statistically significant (*p* = 0.000 at Wilcoxon test); the same conclusion was reached for TBI patients, with a mean decrease of 14.84 (35.8%) points from initial 41.6 points [C.I. 37.29–45.93] to 26.76 [C.I. 21.94–31.59] (*p* = 0.002).

As for HADS scores (range 0–21 points for anxiety, 0–21 depression) a smaller improvement was found in the cohort of SCI patients: the main value for anxiety registered a 1-point decrease at T1 (21.5%), from initial mean 6.5 points [C.I. 3.80–9.34] to 5.1 [C.I. 3.17–7.11]. This variation was near the threshold of significance at paired t-test (*p* = 0.05). Depression domain, instead, improved by 2.35 mean points (37%), from 6.35 initial points [C.I. 3.45–9.26] to final 4 [C.I. 2.15–5.98] with reaching of a significant *p* value at paired t-test (*p* = 0.03).

ANCOVA confirmed no statistical significance in changes in anxiety section of HADS (F = 2.58, *p* = 0.13), adjusting for age and gender; no significant interaction was detected with respect to covariate (age-*p* = 0.750, gender-*p* = 0.17). As for the depression section, results were not confirmed at ANCOVA: pre/post changes were not significant (F = 1.1, *p* = 0.32), but no influence of gender (*p* = 0.26) or age (*p* = 0.08) was retrieved.

**Figure 2:**
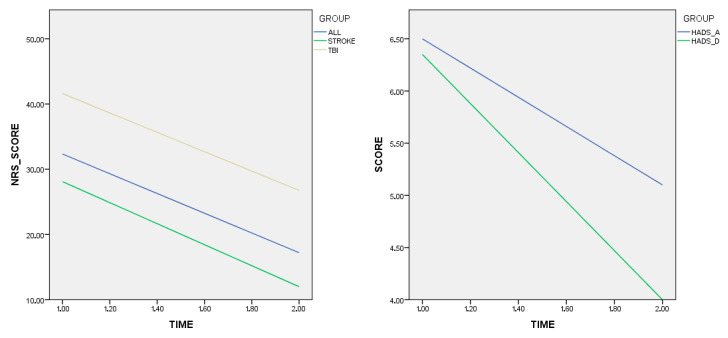
Line plot for NRS and HADS variations in all tested populations; timeline proceeds from 1 (T0) to 2 (T1). Time is represented from T0 (1.00) to T1 (2.00); each interval represents a week.

In Figure 1 and Figure 2, line plots of NRS and HADS variations were reported.

## 4. Discussion

The aim of the present study was to determine if AAA could be effective in a population which, to date, is not well explored by the literature; neurologic patients in an intensive rehabilitation setting. Scientists, in fact, pay greater attention to AAT, maybe because they are intended to support diagnostic/therapeutic applications [21]. In AAT context the animal is seen as a “co-therapist”, actively contributing to the patient’s ability to recover, even in neurologic settings [22,23,24,25,26].

In the AAA perspective, instead, the focus is enhancing the patient’s well-being and mood, and decreasing pain perception, thus reaching a greater care engagement [7,27]. This rationale is particularly attractive for conditions in which a length of stay is usually prolonged, with high effort in terms of engagement and collaboration in physical therapy; all three conditions we studied (spinal cord injury, stroke, traumatic brain injury) share these characteristics [28,29,30,31,32].

Our study, as such, represents one of the first attempts to verify the experience of AAA in neurologic patients with high rehabilitation needs: our analysis, from this point of view, gave first preliminary evidence to this rationale, although some criticalities emerged. Simple groups’ tests, in fact, gave a significant result for NRS variations (stroke and TBI populations), confirmed at ANCOVA; despite this, a significant influence of baseline sample characteristics (excluding gender and age) emerged from our test, limiting generalizability of data. Particularly, severity of disease (measured through specific scales, such as NHI stroke scale, American spinal cord injury association—ASIA score or Level of cognitive functioning scale—LCF) could influence the patient’s ability to participate in the AAA sessions, and as such his/her response to the treatment; in the present study, these data were not included in ANCOVA since they were not included in the protocol approved by ethics committee.

The need to further explore this gap with a structured approach in which all possible confounders are controlled represents the main challenge in reaching consistent evidence for AAA in these populations. If confirmed, this would represent particularly interesting data: having a patient with lower attention deficit or less hostility/ depressed mood means the possibility of easily achieving an active participation in all rehabilitation practices, with significant gains in terms of clinical outcome, length of stay and complexity and discharge [33,34,35,36].

As for SCI patients, although starting with low detected levels of anxiety and depression, the first domain’s changes were close to the threshold but not significant (*p* = 0.05), while depression ones resulted in significance at *t*-test but were not confirmed at ANCOVA. Again, here, ANCOVA reflected the tendency of the sample to be influenced by baseline characteristics during AAA intervention, excluding gender and age (and so a weakness of this finding).

Interpretation of these data suggest that AAA deserve better deepening for early inpatient setting after a spinal cord injury; difference in lesion neurological level, completeness of SCI, level of self-efficacy, stage of elaboration of motor deficit and the need to accept permanent changes in mobility and personal relations could play a central role in modulating anxiety and depression [37,38]. This underlines the need to tailor AAA application for SCI patients, involving them in greater pathways in which psychological intervention precedes animals’ entry by preparing the patient for a positive interaction and giving them coping strategies.

Our findings seem in line with the general judgment of AAA present in the literature as an adjunctive tool to improve mood, decrease stress and depression and enhance quality of life [15,16,17]; with the present study we preliminarily explored a “gap” area, with preliminary findings results. Some limitations emerged in our study: first of all, the number of patients (50) was not completely representative of included populations, since epidemiological burden of spinal cord injury, stroke and TBI is higher [21,39]. Then, the low number of included patients with hemorrhage did not allow us to perform sub-analyses for stroke population, so we do not exactly know which stroke form is most responsive to AAA sessions.

Moreover, the lack of a control group represents a strong limit to generalization of our results, since it represents a limit to generalization and does not allow us to establish definitive causal relationships. Another lack was relative to the impossibility of applying blinding strategies for evaluators (Neuropsychologist and psychologist), although they were not directly involved in conducting the AAA; this introduces a possible risk of observation bias. Finally, in the present study we set a limit of sessions to verify protocol’s effect, but most patients (46 out of 50) continued to attend AAA after T1 evaluation; maybe a further timing of scales administration would have consolidated results.

Future studies should verify our findings on larger samples, deepening in particular the moment in which to propose AAA during inpatient rehabilitation approach, and verifying its effects in the long term. Also, the literature should state the exact number of AAA sessions to reach the best benefit for patients, since our study had no further data than T1; evaluating the time/benefit timeline would clarify the number of resources to be invested in AAA to optimize positive impact.

A further point for future studies should be the conduction of qualitative research in which, beyond the biological effect of the AAA, the patient could recount his experience and return the value of the approach in the context of his illness experience; such studies should be enlarged to caregivers too.

## 5. Conclusions

The present study represents the first preliminary evidence in support AAA as an integrative tool to accompany patients with central nervous system disorders along treatment pathway, improving their engagement and neurobehavioral deficits. Although positive trends emerged from our analysis, future research should further deepen the role of confounders in reducing/implementing the effect of this intervention, with particular attention to severity of disease. The need for larger controlled trials also emerged as central for a further understanding of AAA role in rehabilitation setting; finally, defining highly standardized protocols will allow a better integration of AAA in clinical settings, thus representing a valuable investment in intensive rehabilitation practice.

## Figures and Tables

**Table 1:** Summary of patients’ characteristics. TBI: traumatic brain injury; SCI: spinal cord injury; DoC: disorder of consciousness; SD: standard deviation. Nominal variables were described as absolute frequency and percentage.

Variable	
Sex	
Men	40 (61.54%)
Women	25 (38.46%)
Age	Mean (±SD)
Global	63.13 (±16.706) years
Stroke	63.38 (±17.85) years
TBI	64.18 (±16.70) years
SCI	56.68 (±17.58) years
Diagnosis	
Stroke	28 (43%)
Ischaemic	25
Haemorrhagic	3
TBI	15 (23%)
With DoC	0
SCI	22 (34%)
Traumatic	10
Not traumatic	12
Provenience at admission	
Home	8 (12%)
Emergency department	39 (60%)
Trauma center	11 (17%)
Other rehabilitation unit	7 (11%)
Time by diagnosis	Weeks range
Stroke	2–4 weeks
TBI	7–8 weeks
SCI	20–21 weeks
Functional state (Modified Barthel Index)	
Complete dependence (0–20)	0
Severe dependence (21–60)	13 (20%)
Moderate dependence (61–90)	40 (62%)
Slight dependence (91–99)	10 (15%)
Independence (100)	2 (3%)

**Table 2:** Results of interest variables’ tests. NRS: Neurobehavioral Rating Scale; HADS: Hospital Depression and Anxiety Scale; TBI: traumatic brain injury; SD: standard deviation; IRQ: inter-quartile range; Pts: points. ** value not confirmed at ANCOVA.

Outcome of Interest	Pre-Procedural Score (Mean ± SD; 95% Confidence Interval; Median; IRQ)				Post-Procedural Score (Mean ± SD; 95% Confidence Interval Median; IRQ)				Statistics (*t*-Test/Wilcoxon Test)
NRS Score-TBI + stroke	32.34 (±15.27) pts	[26.83–37.35]	35 pts	27.75	17.21 (±12.61) pts	[12.66–21.76]	14.50 pts	19	*p* = 0.000 (TT)
NRS Score-Stroke	28.10 (±15.66) pts	[20.55–35.65]	22 pts	21	12 (±11.1) pts	[6.6–17.36]	8 pts	12	*p* = 0.000 (WT)
NRS score-TBI	41.6 (±7.1) pts	[37.29–45.93]	41 pts	8	26.76 (±7) pts	[21.94–31.59]	25 pts	10.5	*p* = 0.002 (WT)
HADS-Anxiety	6.5 (±4.79) pts	[3.80–9.34]	6 pts	8.25	5.1 (±3.41) pts	[3.17–7.11]	5.5 pts	5.25	*p* = 0.05 (TT)
HADS-Depression	6.35 (±5.03) pts	[3.45–9.26]	5 pts	9.5	4 (±3.41) pts	[2.15–5.98]	3.5	5.5	*p* = 0.03 ** (TT)

## Data Availability

The original contributions presented in this study are included in the article. Further inquiries can be directed to the corresponding author.

## Abbreviations

The following abbreviations are used in this manuscript:

AAA	Animal-assisted activity
AAT	Animal-assisted therapy
AAI	Animal-assisted intervention
AAE	Animal-assisted education
SCI	Spinal cord injury
TBI	Traumatic brain injury
